# First record of the leafhopper genus *Sweta* Viraktamath & Dietrich (Hemiptera, Cicadellidae,Typhlocybinae) from China, with description of one new species feeding on bamboo

**DOI:** 10.3897/zookeys.187.2805

**Published:** 2012-04-27

**Authors:** Lin Yang, Xiang-Sheng Chen, Zi-Zhong Li

**Affiliations:** 1 Institute of Entomology, Guizhou University, Guiyang, Guizhou, 550025, P.R. China; 2 The Provincial Key Laboratory for Agricultural Pest Management of Mountainous Region, Guizhou University, Guiyang, Guizhou, 550025, P.R. China

**Keywords:** Cicadomorpha, distribution, Oriental region, taxonomy

## Abstract

*Sweta bambusana* **sp. n.** (Hemiptera: Cicadellidae: Typhlocybinae: Dikraneurini), a new bamboo-feeding species, is described and illustrated from Guizhou and Guangdong of China. This represents the first record of the genus *Sweta* Viraktamath& Dietrich from China and the second known species of the genus. The new taxon extends the range of the genus *Sweta*, previously known only from northeast India and Thailand, considerably eastwards. A key for separation of the species of *Sweta* is given.

## Introduction

The leafhopper genus *Sweta* was established by Viraktamath and Dietrich (2011) based on the type species *Sweta hallucinata* Viraktamath & Dietrich, 2011, from northeast India and Thailand. This dikraneurine genus is remarkable because it shares features with another leafhopper subfamily, Signoretiinae, restricted to the Old World tropics (Viraktamath and Dietrich 2011).

During the course of studying species biodiversity of the bamboo-feeding leafhoppers in China (see Discussion), several specimens belonging to an undescribed species of the genus *Sweta* were found. The new species represents the first record of *Sweta* in China, and its discovery has broadened our knowledge of host plant and biogeography of the genus.

## Materials and methods

Terminology used in this work follows Dietrich (2005). Dry specimens were used for the description and illustration. External morphology was observed under a stereoscopic microscope and characters were measured with an ocular micrometer. Measurements are given in millimeters; body length is measured from the apex of the head to the apex of the forewing in repose. The genital segments of the examined specimens were macerated in 10% KOH, washed in water and transferred to glycerine. Illustrations of the specimens were made with a Leica MZ 12.5 stereomicroscope. Photographs of the types were taken with a Leica D-lux 3 digital camera. The digital images were then imported into Adobe Photoshop 8.0 for labeling and plate composition. Nomenclature of leg setae follows Li et al. (2011). The type specimens are deposited in the Institute of Entomology, Guizhou University, Guiyang, China (IEGU), and the Natural History Museum, London (BMNH).

## Taxonomy

### Sweta

Viraktamath & Dietrich, 2011

http://species-id.net/wiki/Sweta

Sweta Viraktamath & Dietrich, 2011: 1.

#### Type species.

*Sweta hallucinata* Viraktamath & Dietrich, 2011, by original designation.

#### Diagnosis.

Small size. Crown of the head strongly elevated above the anterior margin of the pronotum. Ocelli vestigial. Pronotum enlarged, strongly convex, and extended to the scutellar suture. Forewing broad, tectiform, with elongate, sinuate distal segments of veins R and M, closed preapical cells absent. Hind wing with the submarginal vein complete and veins RP and MA confluent. First hind tarsomere acuminate. Aedeagus fused to the connective. Female second valvulae asymmetrical.

#### Distribution.

Oriental region (Fig. 19).

#### Remarks.

This dikraneurine genus is remarkable because it has a feature unknown in other typhlocybinae but present in another leafhopper subfamily (Signoretiinae), i.e. the elongate pronotum. A full description of the genus was given by Viraktamath and Dietrich (2011).

#### Key to species of *Sweta* Viraktamath & Dietrich (male)

**Table:** 

1	Aedeagus with lower preapical processes distinctly longer than more distal processes; male abdomen with 3S apodemes extended to midlength of segment IV	*Sweta hallucinata*
–	Aedeagus with lower preapical processes slightly shorter than more distal processes (Figs 9–11); male abdomen with 3S apodemes extended to midlength of segment V (Fig. 5)	*Sweta bambusana* sp. n.

### Sweta bambusana sp. n.

urn:lsid:zoobank.org:act:E0CB16F2-4D98-4080-8981-9D69A313A2CF

http://species-id.net/wiki/Sweta_bambusana

Figs 1 16

#### Type material.

Holotype: ♂, **China:** Guizhou, Huishui, Yantang (26°08'N, 106°39'E), collected from bamboo (*Dendrocalamus affinis*), 31 May 2008, X.-S. Chen and L. Yang (IEGU); paratypes: 3 ♀♀, same data as holotype (IEGU); 3 ♂♂, 4♀♀, Guizhou, Changshun, Weiyuan (26°02'N, 106°27'E), collected from bamboo (*Dendrocalamus affinis*), 30 Sept. 1997, X.-S. Chen and L. Yang (IEGU), one male and female deposited in BMNH; 3 ♀♀, Guangdong, Guangzhou, Baiyunshan (23°10'N, 113°18'E), collected from bamboo, 21 Nove. 2006, X.-S. Chen (IEGU).

#### Etymology.

The new species is named after the host plant bamboo (Bambusoideae).

#### Description.

Body length (from apex of vertex to tip of forewings): male 4.03–4.15 mm (N = 5); female 3.75–4.22 mm (N = 10); forewing length: male 3.25–3.30 mm (N = 5); female 3.05–3.31 mm (N = 10).

#### Coloration.

Milky white to pale yellow (Figs 13-16). Forewing cells rather clouded with faint pale brown; distal portions of tarsi dark brown (Figs 3, 13, 14).

#### Head and thorax.

External features as in diagnosis. Crown shorter medially than width between eyes (0.14:1). Pronotum shorter medially than width at widest part (0.55:1), longer medially than crown (4.0:1). Scutellum shorter medially than pronotum (0.31: 1). Forewing longer medially than width at widest part (2.95:1). Hindwing longer medially than width at widest part (3.65:1).

#### Abdomen.

Male abdomen with 3S apodemes subparallel, extended to midlength of segment V (Fig. 5).

#### Male genitalia.

Aedeagus with both pair of preapical processes curved laterally, lower pair slightly shorter than more dorsal pair (Figs 9, 10, 11); shaft apex blunt and rounded. Other features as in generic diagnosis.

#### Female genitalia.

Seventh sternite (Fig. 12) broad basally, triangularly produced posteriorly with rather rounded apex.

#### Host plant.

Bamboo (*Dendrocalamus affinis* (Rendle) Mcclure) (Figs 17, 18).

**Figures 1–12.**
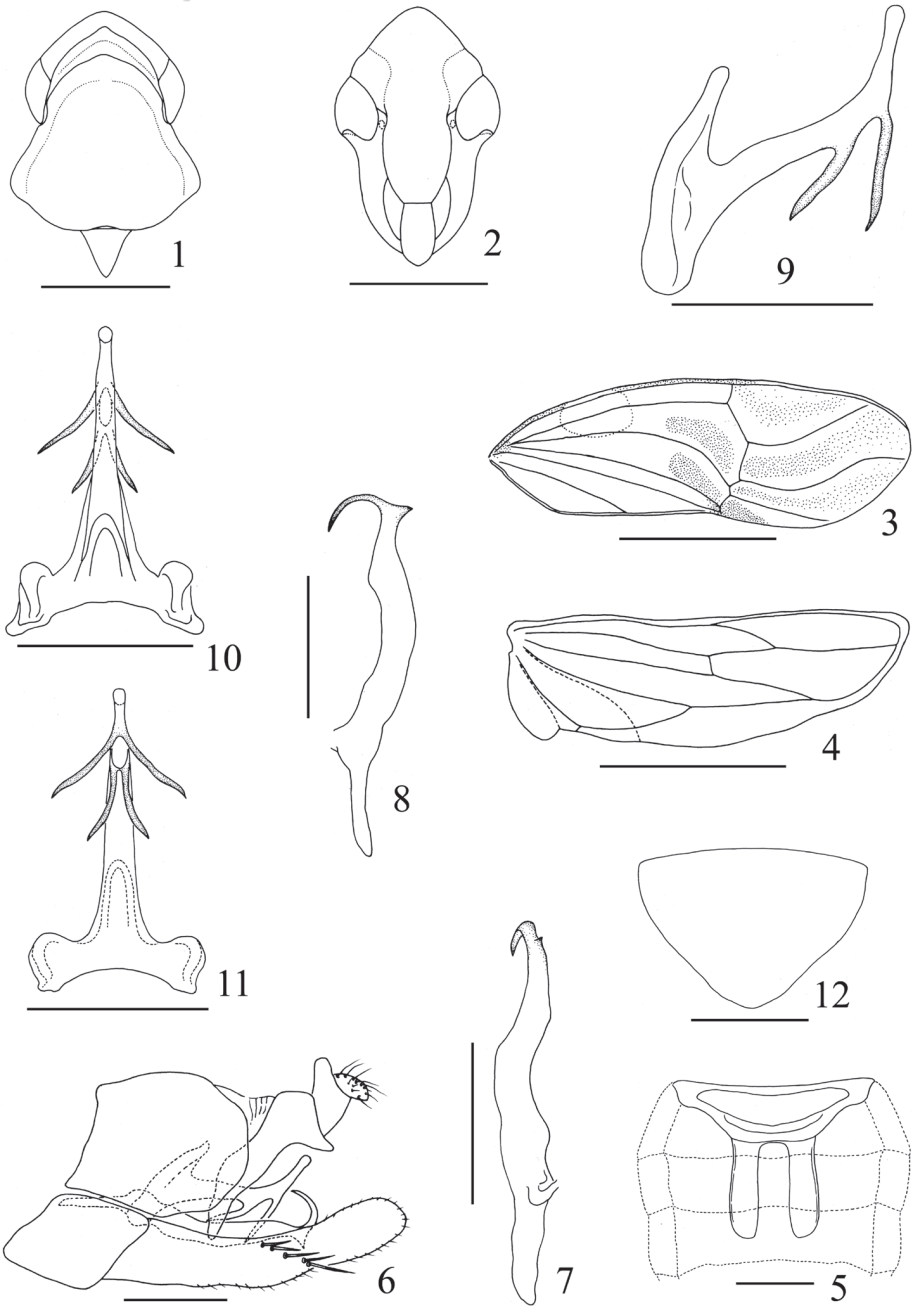
*Sweta bambusana* sp. n. **1** Head and thorax, dorsal view **2** Head, anteroventral view **3** Forewing **4** Hindwing **5** Base of abdomen, ventral view **6** Male genital capsule, lateral view **7** Style, ventral view **8** Style, lateral view **9** Aedeagus, lateral view **10** Aedeagus, dorsal view **11** Aedeagus, ventral view **12** Female abdominal sternite VII, ventral veiw. Scale bars: = 1 mm (Figs 3, 4), 0.5 mm (Figs 1, 2), 0.2 (Figs 5–12)

**Figures 13–16.**
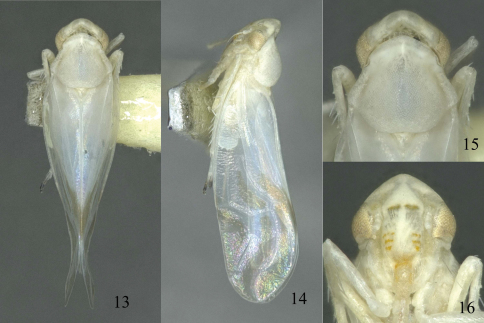
*Sweta bambusana* sp. n. **13** Dorsal habitus, holotype from Huishui **14** Lateral habitus, holotype from Huishui **15** Head and thorax, dorsal view **16** Head, anteroventral view.

**Figures 17–18.**
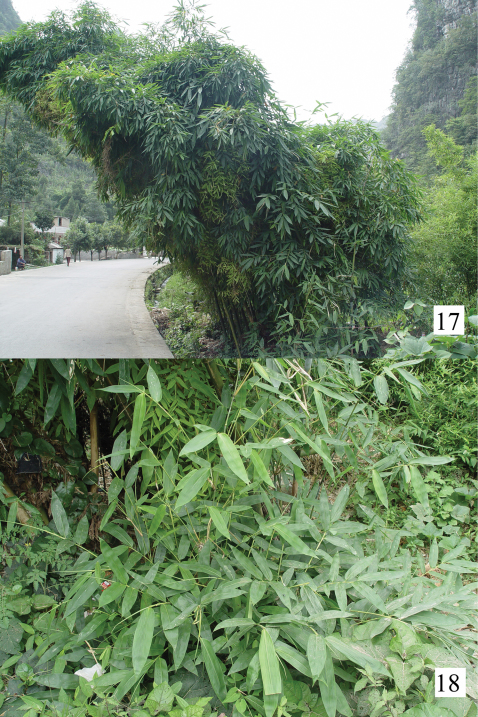
Host plant of *Sweta bambusana* sp. n. **17** View of the area where the types of *Sweta bambusana* were captured, in Changshun (Guizhou, China) with *Dendrocalamus affinis* **18** View of the plant.

**Figure 19.**
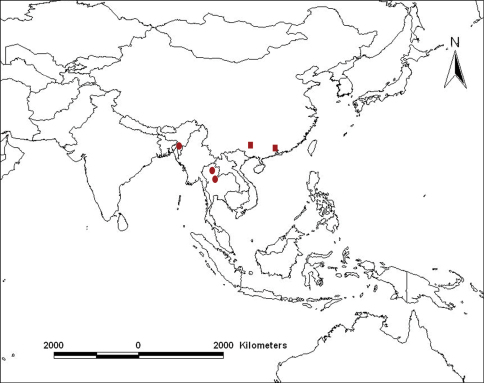
Geographic distribution of *Sweta* species: *Sweta hallucinata* (●); *Sweta bambusana* sp. n. (■).

#### Distribution.

Southern China (Guizhou and Guangdong) (Fig. 19).

#### Remarks.

This new species is very closely related to *Sweta hallucinata* Viraktamath & Dietrich, 2011 from Thailand and India, but can be distinguished by the aedeagus with apex blunt and rounded (tapering in *hallucinata*); the lower pair of subapical processes slightly shorter than more dorsal pair (in *hallucinata*, the lower pair are distinctly longer than the more upper pair); male abdomen with the 3S apodemes extended to midlength of the segment V (extended to midlength of the segment IV in *hallucinata*); the female abdominal sternite VII more or less triangular (relatively rounded in *hallucinata*).

## Discussion

**Diversity of bamboo-feeding leafhoppers.** The present authors have paid particular attention to the species of bamboo-feeding leafhoppers in their field research and collected large numbers of specimens in the past 15 years from China including a number of new taxa and new records (Chen and Li 1997; Li and Chen 1999; Chen et al. 2007, 2008, 2009; Yang et al. 2007; Li et al. 2007, 2011; Yang and Chen 2011). Yang et al. (1999) recorded 13 species within 9 genera from China and Yang et al. (2011) increased the number to 33 genera and 55 species (in 9 subfamilies), of which, 4 species belonged to Typhlocybinae. Clearly, throughout China, the species diversity of bamboo-feeding leafhoppers is very great with more than 87 species feeding exclusively on Bambusoideae (Chen et al. in press).

**Host plant of new species.** *Sweta bambusana* was found feeding exclusively on one species of native bamboo, *Dendrocalamus affinis* (Rendle) McClure (Figs 17, 18). No other information on biology of *Sweta* species, nor host plant damage caused, is available.

**Distribution of *Sweta* species.** Although both species of *Sweta* appear to be widespread in Southeast Asia (Fig. 19) they are very rare. The new species extends the range of the genus eastwards from northeast India and Thailand to China.

## Supplementary Material

XML Treatment for Sweta

XML Treatment for Sweta bambusana
